# Congenital Unilateral Hypoplasia of Depressor Anguli Oris

**DOI:** 10.1155/2012/507248

**Published:** 2012-09-17

**Authors:** Seckin O. Ulualp, Ronald Deskin

**Affiliations:** Division of Pediatric Otolaryngology, Department of Otolaryngology-Head and Neck Surgery, University of Texas Southwestern Medical Center and Children's Medical Center, 5323 Harry Hines Boulevard, Dallas, TX 75390-9035, USA

## Abstract

*Objectives*. Asymmetric facial appearance may originate from abnormalities of facial musculature or facial innervation. We describe clinical features of congenital hypoplasia of depressor anguli oris muscle in a child. *Material and Methods*. Chart of a 10-month-old female referred to a tertiary care pediatric hospital for assessment of facial paralysis was reviewed. Data included relevant history and physical examination, diagnostic work up, and management. *Results*. The child presented with asymmetric movement of lower lip since birth. Asymmetry of lower lip was more pronounced when she smiled and cried. Rest of the face movement was symmetric. On examination, the face appeared symmetric at rest. The child had inward deviation of right lower lip when she smiled. Facial nerve function, as determined by frowning/forehead, wrinkling, eye closure, nasolabial fold depth, and tearing, was symmetric. Magnetic resonance imaging of the temporal bones and internal auditory canals were within normal limits. Echocardiogram did not show cardiac abnormality. Auditory brainstem response showed no abnormality. *Conclusions*. Congenital hypoplasia of depressor anguli oris is a rare anomaly that causes asymmetric crying face. Pediatricians and otolaryngologists need to be cognizant of cardiac, head and neck, and central nervous system anomalies associated with congenital unilateral hypoplasia of depressor anguli oris.

## 1. Introduction

Congenital unilateral hypoplasia of depressor anguli oris has been implicated in the pathogenesis of asymmetric crying face since Parmalee first described facial weakness apparent during crying in 1931 [1]. Asymmetric face is estimated to occur in 0.2%–0.6% of infants [2, 3]. Clinical presentation of children with asymmetric crying face is characterized by drooping of one corner of mouth on the intact side while crying. Diagnosis can be established by the clinical picture and/or an electromyographic study [4]. A wide variety of anomalies involving cardiovascular, gastrointestinal, genitourinary, skeletal, and central nervous systems may be seen in children with congenital hypoplasia of depressor anguli oris [3, 5–10]. Accurate diagnosis of this subtle condition ensure proper management and screening for associated anomalies. This retrospective case review describes clinical features of congenital hypoplasia of depressor anguli oris muscle in a child.

## 2. Case Report

**Figure 1:**
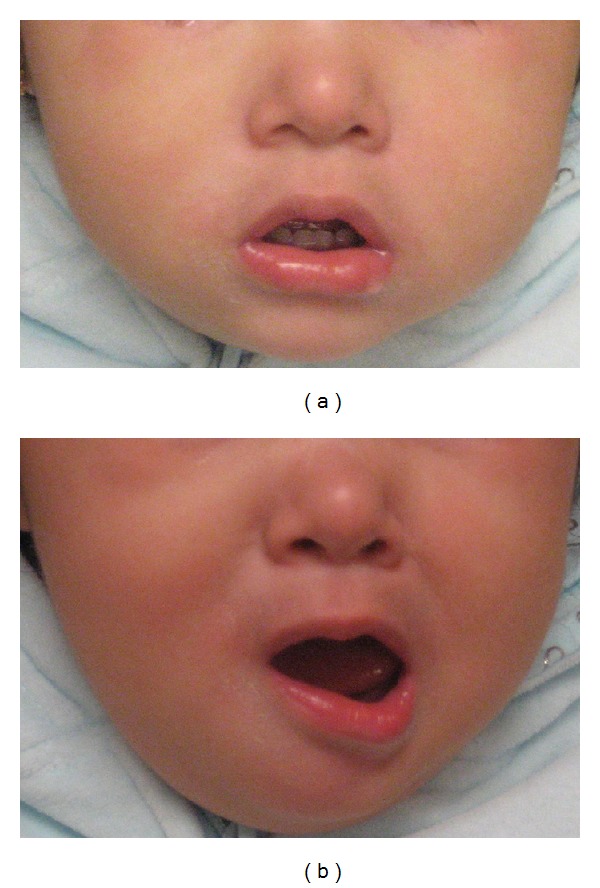
Child with right-sided asymmetric crying face at rest (a) and crying (b). The lower lip is pulled toward the intact left side.

A ten-month-old Hispanic girl presented to the pediatric otolaryngology clinic for evaluation of asymmetric face. Mother noticed asymmetric movement of lower lip since birth. Asymmetry of lower lip was more pronounced when she smiled and cried. Rest of the face movement was symmetric. The child was born full-term with no complications to a mother without medical problems. Past medical history and family history were unremarkable. Physical examination revealed well-appearing child in no respiratory distress, normal otologic exam, normal nasal exam, and normal oral cavity exam. The face appeared symmetric at rest. The child had inward deviation of right lower lip when she smiled (Figure 1). Facial nerve function, as determined by frowning/forehead, wrinkling, eye closure, nasolabial fold depth, and tearing, was symmetric. Neurodevelopmental exam was normal. Magnetic resonance imaging of the temporal bones and internal auditory canals were within normal limits. Echocardiogram did not show cardiac abnormality. Auditory brainstem response test showed no abnormality.

## 3. Discussion

Congenital unilateral hypoplasia of depressor anguli oris causes congenital asymmetric crying face. Typical clinical presentation of children with congenital unilateral hypoplasia of depressor anguli oris includes typical clinical picture, which includes lower lip asymmetry during crying while forehead wrinkling, nasolabial fold depth, and eye closure remain intact and equal on both side [4]. Differential diagnosis of asymmetric crying face includes facial nerve paralysis and obstetric-related compression or trauma factors. Facial nerve lesions may cause weakness of facial expressions; however, congenital hypoplasia of depressor anguli oris afflicts lower lip while other functions of facial nerves are preserved. Diagnosis can be established by the clinical picture and/or an electromyographic study. In previous studies, electromyography has confirmed the myogenic nature of this condition [11, 12]. Functional prognosis of outcome congenital unilateral hypoplasia of depressor anguli oris has been poor. 

 Coexisting congenital anomalies may occur in 20%–70% of children with asymmetric crying face [13–16]. The wide range of occurrence appears to be due to size of the study group and patients characteristics such as inclusion of patients with variety of syndromes. Children with asymmetric crying face may have coexisting Cayler cardiofacial syndrome, velocardiofacial syndrome, CATCH 22 (cardiac defect, abnormal facies, thymic hypoplasia, cleft palate, and hypocalcemia), VACTERL (vertebral anomalies, anal atresia, cardiac malformations, tracheoesophageal fistula, renal anomalies, limb abnormalities), and Trisomy 18. Cardiovascular system and head and neck anomalies have been the most common anomalies. Skeletal, genitourinary, central nervous system, gastrointestinal anomalies are less frequently observed. A wide variety of cardiac anomalies, including but not limited to, ventricular septal defect, tetralogy of Fallot, patent ductus arteriosus, coarctation of the aorta, and atrial septal defects may occur. Cardiac anomalies increase the mortality and morbidity. Nearly half of the patients with asymmetric face and cardiac anomalies developed congestive heart failure [5]. Head and neck anomalies associated with asymmetric face encompass auricular malformation, maxillary/mandibular hypoplasia, low set ears, and auditory dysfunction [16]. Our patient did not have coexisting cardiac anomalies as documented by echocardiogram. Magnetic resonance imaging did not document abnormalities of the facial nerve. Auditory brainstem response test confirmed the intact function of hearing pathway. 

The pathogenesis of congenital unilateral hypoplasia of depressor anguli oris has not been established. To date, intrauterine molding, subclinical viral infection during pregnancy, and heredity have been suggested as causative factor [5, 12, 17]. Nonetheless, pathogenesis of congenital unilateral hypoplasia of depressor anguli oris appears to be multifactorial and further studies are needed to elucidate it. 

In conclusion, the present case highlights the clinical presentation of congenital unilateral hypoplasia of depressor anguli oris that causes asymmetric crying face. Pediatricians and otolaryngologists need to be cognizant of cardiac, head and neck, and other system anomalies associated with asymmetric crying face. Combination of high clinical suspicion and thorough search for abnormalities in other systems ensures early diagnosis, proper management, and prevention of complications in children with asymmetric crying face.
